# Rethinking evolutionary inference in metagenomic time series

**DOI:** 10.1128/msystems.00693-26

**Published:** 2026-08-12

**Authors:** Alexander Eiler

**Affiliations:** 1 Centre for Biogeochemistry in the Anthropocene, Department of Biosciences, University of Oslo 208408, Oslo, Norway; CNRS Delegation Bretagne et Pays de Loire, Nantes, France

**Keywords:** eco-evolution, metagenomics, time-series, sweeps, dispersal, environmental sorting

## Abstract

As ecologists increasingly use metagenomic time series to track evolution in the wild, there is a risk of misinterpreting ecological dynamics as rapid adaptation. This Perspective identifies methodological limitations that generate misleading signatures of microbial evolution. A primary issue is confusing evolutionary change (driven by *de novo* mutation or horizontal gene transfer) with ecological lineage turnover, such as seasonal oscillations or the reactivation of dormant lineages. Current metagenome-assembled genomes can collapse micro-diverse lineages and decouple adaptive mobile elements, creating inaccurate genomic signatures of sweeps or stasis. To address these issues, I propose a framework integrating long-read sequencing, pangenome graph theory, and forward-time simulations to model populations as temporal genetic networks and better resolve microbial evolutionary dynamics.

## PERSPECTIVE

A common expectation in modern microbiome research is that microbes evolve rapidly (1). Whether in response to pollution, climate change, or host interaction, it is often assumed that high turnover rates and large population sizes facilitate the observation of adaptation in real-time (2). However, a discrepancy exists between theoretical expectations, which are often derived from closed-system chemostats, and recent findings from metagenomic time-series data (3, 4).

**Fig 1:**
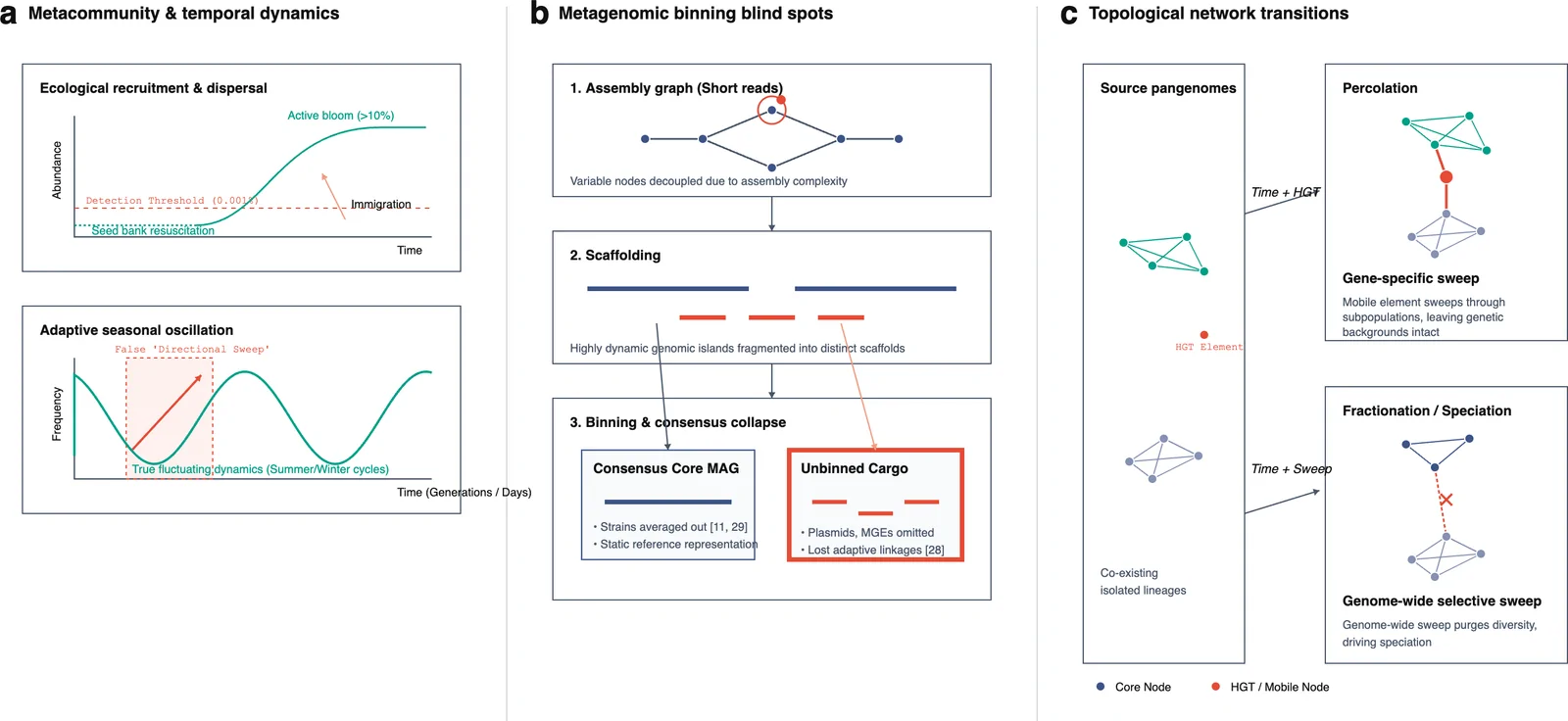
Directionality and the topological resolution. (**a**) Putting metagenomic data into a metacommunity context can reveal ecological drivers: (i) the reactivation of dormant strains from a local seed bank, and the immigration of pre-existing lineages from interconnected environments; and (ii) seasonal oscillation, where directional selection represents a phase of a long-term cycle. (**b**) Current metagenomic pipelines reconstruct composite genomes (MAGs), where assembly graphs are broken into scaffolds that are subsequently binned into a consensus core often omitting accessory cargo such as genomic islands. (**c**) The dynamic graph theory, treating the pangenome as a temporal network. Evolution is detected via topological events, such as percolation (gene flow connecting populations) and fractionation (speciation via network breakage), where the latter may be the result of a genome-wide selective sweep. Colors indicate different populations (or “species”).

Analyses of long-term microbial data sets have begun to expose limitations in studying evolution via metagenomic time-series (5, 6). A primary challenge is that newly detected genomes are sometimes mistaken for evolutionary events, when they may actually represent rare, undetected genotypes increasing in abundance, a process known as ecological recruitment or ecological lineage turnover (Fig. 1a). Other studies have shown that while genome-wide selective sweeps occur (7), natural systems are dominated by gene-specific selective sweeps (3, 8). This Perspective examines these methodological limitations in interpreting open-system evolutionary studies and proposes a revised framework for analyzing metagenomic time-series data.

## DISTINGUISHING GENETIC NOVELTY FROM ECOLOGICAL TURNOVER

Interpreting metagenomic time series requires distinguishing the formation of genetic novelty from ecological lineage turnover (9, 10), also termed ecological strain turnover. Here, ecological turnover is defined as the shift in relative abundance of distinct, pre-existing lineages within a community. This is different from a novel variant driving allele-frequency shifts, via *de novo* mutation, recombination, or horizontal gene transfer (HGT). In this context, a population is defined as an ecologically and genetically cohesive group of individuals (or lineages), maintained by recent common ancestry and sufficient homologous recombination to prevent divergence (11).

When environmental conditions favor one distinct lineage over another, ecological turnover manifests as a sudden, massive shift in allele frequencies within the population. This shift can be misinterpreted as a rapid evolutionary sweep. In closed laboratory systems, a lineage rising from zero to dominance is correctly interpreted as a selective sweep of a *de novo* variant (12). Natural ecosystems, however, function as an open system with massive seed banks of standing variation (13–15). In population genetics, standing variation is strictly defined as the presence of alternative alleles or distinct genetic variants within a population prior to a shift in environmental conditions or selection pressures (16). Thus, a specific strain may persist at undetectable abundances (<0.001%) within the rare biosphere or be introduced via continuous dispersal from linked systems, only to be rapidly recruited into the dominant community (exceeding 10% relative abundance) when environmental conditions change (13, 17, 18) (Fig. 1a). When standard read-mapping approaches are used to identify sweeps, this rapid ecological turnover of a pre-existing strain can be most easily confused with genome-wide sweeps, and less likely with gene-specific sweeps (19).

To overcome such misinterpretations, a framework that explicitly differentiates ecological turnover and evolutionary selection is needed. Biologically, these processes are linked: the standing variation recruited today is simply the legacy of evolutionary events from the past. Yet, to estimate contemporary evolutionary rates, the reshuffling of existing diversity (ecological turnover) must be distinguished from the selection on genetic novelty resulting from *de novo* mutation and recombination.

While these two processes operate at fundamentally different biological levels, they can produce indistinguishable signatures in standard metagenomic analyses. Evolutionary change is driven by mutation, recombination, or HGT, and shifts allele frequencies within a single, recombining population. Ecological turnover, conversely, shifts the relative abundances of existing genetic lineages within a broader community (20, 21). Because standard metagenomic binning often collapses distinct, co-existing lineages into a single composite MAG, the ecological turnover of lineages (a shift in their relative abundance) manifests bioinformatically as a sudden appearance of a clonal novelty. Conflating the turnover of existing diversity with the generation of genetic novelty can overestimate evolutionary rates.

Without accounting for immigration and the broader metacommunity, local evolution models may yield inflated mutation and recombination rate estimates. While immigration introduces novel SNPs that inflate apparent mutation rates, the co-occurrence of these divergent immigrant lineages (admixture) also severely confounds recombination estimates. Because immigrant strains originate from the broader metapopulation, they introduce the genetic legacy of a vastly larger effective population size into the local scale. Consequently, unresolved admixture creates mosaic genomic signatures that local models easily misinterpret as rapid *in situ* recombination (8). Hence, validating local evolution requires cross-referencing new lineages against global data sets to account for this metacommunity dispersal (22, 23). Since it is not possible to cover all microbial lineages across time and space, novelty must be assessed cautiously, with the null hypothesis remaining the establishment of a dispersed lineage rather than local adaptation.

In classical deterministic evolutionary models, the ecological recruitment of an existing lineage is mathematically indistinguishable from a genome-wide sweep of a *de novo* lineage (16). Even in sophisticated Bayesian frameworks (24), without accurate priors on the rare biosphere, the model may erroneously attribute ecological turnover to the acquisition of genetic novelty. That a pre-existing lineage was merely selected from the standing stock, and failing to correct for this resuscitation effect, can also lead, though less likely, to an overestimation of gene-specific sweeps (19).

## SEASONAL OSCILLATION VERSUS ARMS RACE DYNAMICS

Extrapolating from the predictable dynamics of closed-system laboratory experiments (1, 2), a common expectation when analyzing decadal metagenomic time-series is to look for directional selection toward a new evolutionary optimum (e.g., in response to progressive climate change). This view can overlook the biphasic adaptive landscape of temperate ecosystems, where selection pressures fluctuate seasonally (25). For example, in a hypothetical 15-year data set (~2,500 generations approximated from a time series of ~5,000 days with a doubling of cells every second day) might capture a lineage oscillating between two fitness peaks. This could appear as noise in short-term studies or as a directional sweep if the observation window is too narrow.

As Ward et al. (26) demonstrated, selection pressures in these systems often do not persist long enough to drive a species to a permanent optimum; traits favored in July become fitness liabilities in January. This presents a challenge for interpretation in short-term studies. Rapid turnover exposes the aliasing effects inherent in low-resolution time series, where a sampling frequency lower than the rate of environmental fluctuation causes high-frequency biological oscillations to appear as trends, and thus connecting unrelated data points into a false narrative. Apparent rapid, directional sweeps may instead represent the upswing of a long-term oscillation (Fig. 1a), termed adaptive oscillation (25).

Interpreting a net evolutionary distance of zero in decadal time series requires caution as well. A population may appear to be running in place regarding core metabolic traits, creating an appearance of stasis. While abiotic adaptations often oscillate, biotic pressures, such as viral predation drive Red Queen-style evolution (27, 28). Under these dynamics, accessory gene content, such as anti-phage defense mechanisms, undergoes rapid turnover, being continuously gained and lost. Failing to distinguish these signatures in the genomes can create an appearance of rapid evolution in oscillating communities, or inaccurately inferred stasis when arms races are obscured by seasonal fluctuations or methodological limitations.

## DECOUPLING THE CORE FROM THE ACCESSORY GENOME

A key methodological limitation lies in the reliance on MAGs. MAGs are consensus sequences, composites of abundant populations rather than precise clonal lineages (11, 29, 30). They inherently smooth over the variation one seeks to measure (3, 31). Although contemporary metagenomic assemblers successfully reconstruct mobile elements and genomic islands, standard binning algorithms struggle to link the adaptive accessors to the host genome (Fig. 1b). Because genomic islands, such as phage defense systems, exhibit divergent k-mer frequencies and independent coverage fluctuations, they are frequently separated during the binning process rather than being successfully clustered with the stable core genome (29, 31).

While the field has developed sophisticated read-mapping tools (e.g., inStrain [32]) to quantify microdiversity and calculate nucleotide diversity beyond the consensus MAG, these approaches still rely on mapping to a reference scaffold. They remain limited in tracking the dynamics of unbinned genomic islands. Applying standard metrics like FST or dN/dS to composite genomes forces observations into evolutionary models that assume a single, cohesive population. This is particularly problematic for metrices explicitly designed for differences between divergent species, not for segregating polymorphisms within a single population (33). Furthermore, frequency changes among multiple micro-diverse strains (11, 34), that the assembler and binner collapsed into a single MAG, might be interpreted as an allele driving a genome-wide selective sweep through the population.

Long-term evolutionary stasis is a genuine biological phenomenon in certain highly restricted niches (e.g., deep subsurface [35] or obligate endosymbionts [36]). Stasis does not imply a complete disappearance of mutation and HGT; rather, it describes a regime dominated by strong purifying selection that maintains highly conserved genotypes and phenotypes over long periods. However, inferring this adaptive stasis in open environments like the surface ocean is highly prone to methodological error. Focusing evolutionary analyses on the conservatively binned core scaffolds while failing to link the highly dynamic, unbinned genomic islands (37) can inadvertently create an appearance of evolutionary stasis in oligotrophs (31). Single cell sequencing has shown that abundant marine bacteria have a stable core and highly dynamic accessory regions (38, 39). Although metagenomics can recover variable islands (40), standard MAG pipelines routinely miss these linkages.

## GENOME-WIDE VERSUS GENE-SPECIFIC SELECTIVE SWEEPS

Distinguishing between genome-wide and gene-specific selective sweeps is necessary. A genome-wide sweep can result from strong positive selection acting on a clonal lineage (41), but it can also be driven by neutral demographic processes such as founder effects or transmission bottlenecks (8, 42). In contrast, gene-specific selective sweeps can occur when a highly beneficial mobile element (e.g., a viral defense system or metabolic cassette) spreads through a recombining population via HGT without triggering a genome-wide purging of diversity (43, 44). Gene-specific sweeps seem to be more common in nature (3, 43), although their relative frequency compared with genome-wide sweeps remains an active area of debate.

As pointed out earlier, the signature of ecological lineage turnover, where a newly dominant strain replaces an existing one, can mimic a genome-wide clonal sweep and vice versa. In contrast, as recent literature indicates (19), gene-specific sweeps are frequently and robustly inferred in natural populations. Gene-specific sweep can decouple genomic signals: the core genome remains in stasis (suggesting no evolution), while the flexible genome undergoes rapid turnover (37, 44). Still, current metagenomic pipelines that bin reads into consensus MAGs can miss this dynamic entirely, as they either discard the variable regions (where the sweep is happening) or average them out (29, 45). Tracking only the core scaffolds can lead to false conclusions of stasis. Conversely, studies focusing solely on the mobilome (e.g., viromics or plasmid sequencing) observe high fluidity, potentially overestimating the rate of turnover for the population as a whole. The genomic decoupling hypothesis (44), suggests that both states exist simultaneously. The core genome remains in stasis to preserve metabolic integrity, while the flexible genome undergoes fluid turnover to navigate ephemeral biotic pressures. This variation in evolutionary pace across the genome has been highlighted by single-cell studies of oligotrophic (38, 39, 46) and copiotrophic (8) bacteria. Acknowledging variable evolutionary paces across the genome allows the complexity of evolution to be addressed using modeling approaches (43, 44).

This genomic decoupling can have profound implications for the functional dynamics of the community. The rapid gain and loss of the accessory genes, such as the acquisition of metabolic cassettes for novel nutrient exploitation or viral defense islands during intense phage predation, can drive functional shifts within ecosystems. These functional adaptations can sweep through the community while the taxonomic composition of the core genome remains highly stable (3, 37, 44). Standard MAG pipelines routinely discard this functional variability as unbinnable noise (29, 30), and thus fail to monitor the flow of specific functional genes across distinct lineages over time. This can lead to underestimating the true functional dynamics of the microbiome when relying on MAG data.

## CHALLENGES IN MODELING

Simultaneously modeling ecology and evolution presents a major challenge. The current trend toward hierarchical state-space models is particularly risky (47). While conceptually appealing, these data-hungry frameworks try to resolve hidden evolutionary parameters from sparse time-series data, leading to severe over-parameterization (24, 48). The models risk attributing stochastic variance to selection simply because the equations assume that evolution is the primary driver (47).

To prevent over-attributing variance to evolution, researchers can adopt a stepwise null modeling approach, where a neutral ecological model is first fitted to the data (49), and then complex evolutionary models are applied. Only the variance (or residuals) that cannot be explained by the pure ecological processes, should be passed on for evolutionary inference. These evolutionary models need to go beyond assumptions of constant selection coefficients to incorporate time-varying parameters that reflect the oscillating nature of the environment. Additionally, basic phylogenetic inference tools often attempt to reconstruct strictly clonal, “Backward-Time” trees, which struggle to accurately accommodate the high rates of homologous recombination inherent to open microbial systems.

## A METHODOLOGICAL ROADMAP FORWARD

Accurately describing evolution requires moving beyond the assumption that a MAG represents a cohesive evolutionary unit. As mentioned, the current reliance on short-read binning creates an artifactual view of stasis by leaving the hyper-variable regions where adaptation actually occurs unbinned. Transitioning to haplotype-resolved long-read metagenomics (e.g., PacBio HiFi, Nanopore) can span high-entropy regions (50) and links adaptive elements (e.g., viral defense islands, metabolic cassettes) to the conserved scaffolds (core genome). This approach differentiates whole-genome replacements from the lateral acquisition of specific genes. Researchers can track gene flow rather than arbitrary taxonomic bins predefined by identity thresholds (e.g., 95% ANI). Tracking recombination over time enables the detection of instances where boundaries remain constant but population structure shifts due to gene-specific sweeps. It also identifies expansions of the recombination network due to acquired traits or fractionation into distinct cohesive units in response to niche partitioning (Fig. 1c).

It is important to emphasize that traditional reference-based read-mapping and alignment-based networks remain foundational for disentangling eco-evolutionary dynamics. These approaches have driven significant recent discoveries, successfully identifying sub-species populations delimited by gene flow boundaries (51) and providing compelling evidence for gene-specific selective sweeps in natural microbiomes (19). However, I propose pangenome variation graphs as a necessary complementary tool (52, 53). While reference-based methods excel at mapping core-genome microdiversity, they are inherently limited when dealing with highly divergent structural variants or unbinned, orphaned accessory elements. By treating the microbiome as a dynamic network rather than a collection of linear sequences, we can analyze structural dynamics of the pangenome (54, 55) (Fig. 1c).

In this framework, a gene-specific sweep is no longer just a change in abundance but is re-conceptualized as a percolation event: the moment a specific genetic path connects previously fragmented components of the graph (55). This is the moment a specific adaptive gene bridges previously fragmented subpopulations, effectively homogenizing the population at that locus (Fig. 1c). Conversely, topological fractionation signals speciation before ANI divergence occurs. This is where the graph separates into discrete clusters due to barriers in gene flow (11, 56). Perhaps the greatest advantage of graph theory is that it helps resolve the “illusion of directionality.” While the abiotic environment (temperature) may cause the graph to oscillate between two states (fluctuating maintenance), biotic pressures (Red Queen dynamics) will drive the continuous expansion of the graph’s accessory regions (Fig. 1c), thereby capturing these evolutionary dynamics. Differentiating these topological signals allows us to separate the running in place of seasonal adaptation from the network expansion driven by, for example, Red Queen dynamics.

**Fig 2:**
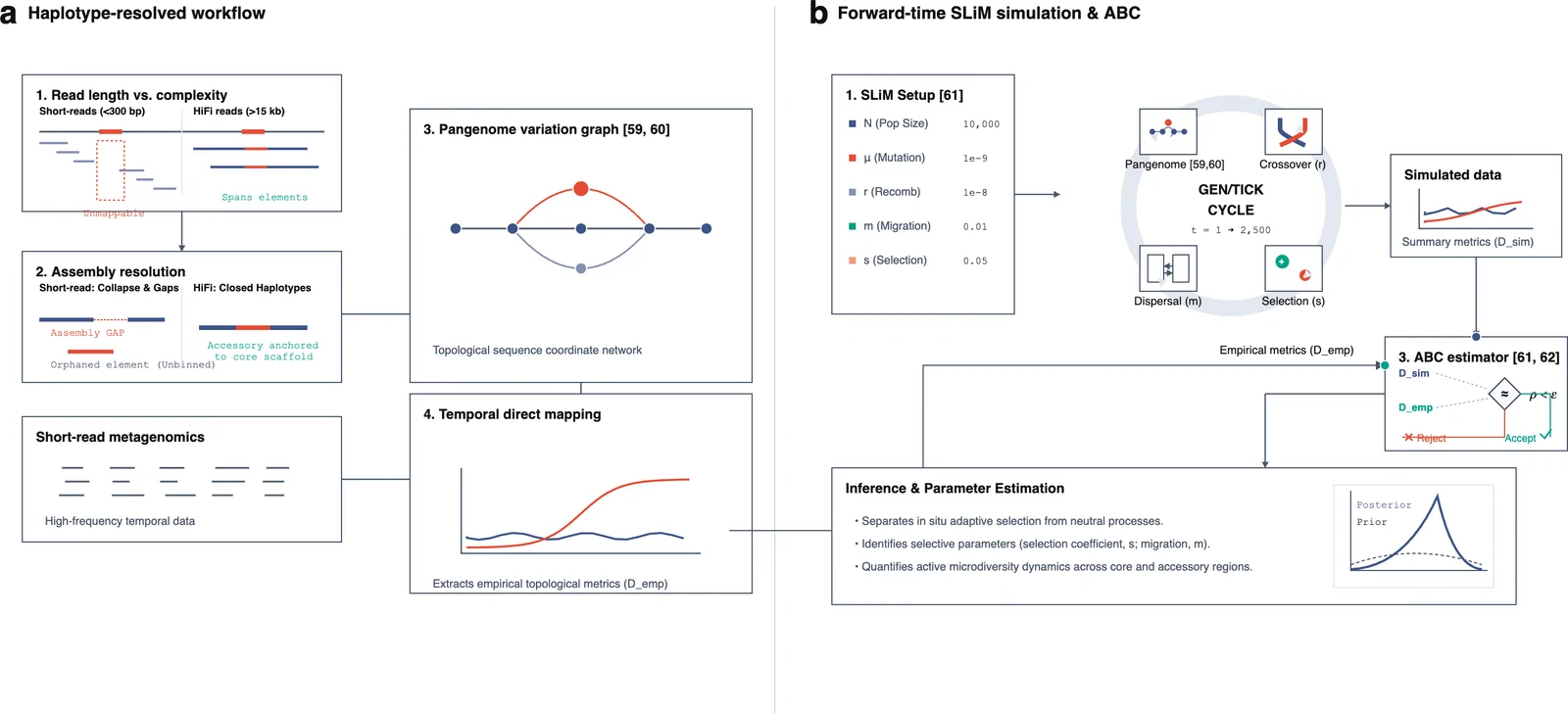
A methodological roadmap. (**a**) The proposed bioinformatic workflow. Long-read sequencing generates haplotype-resolved contigs that retain accessory elements, bypassing the limitations of short-read binning. These contigs are aligned into a pangenome variation graph, creating a unified topological network (59, 60). Read data are then mapped directly onto this graph to quantify temporal coverage across specific nodes and edges. (**b**) The hypothesis-testing framework. Empirical data derived from the pangenome graph are compared against explicit null distributions generated by forward-time simulators (e.g., SLiM) (64). By simulating both ecological lineage turnover (driven by seed banks and immigration) and true evolutionary adaptation (e.g., HGT), approximate bayesian computation (ABC) (64, 65) provides a robust statistical framework to infer the true drivers of observed genomic shifts.

Implementing this framework requires a shift in bioinformatic workflows (Fig. 2a). First, long-read assemblers (e.g., metaFlye [57], HiCanu [58]) need to be applied to generate haplotype-resolved contigs, explicitly bypassing standard short-read binning steps that neglect accessory elements. Second, these contigs should be aligned into a unified topo and thus miss to monitorlogical network using pangenome graph tools (e.g., vg [59] or minigraph [60]), rather than forcing them into a single linear consensus MAG. A limitation of relying on haplotype-resolved long-read assemblies is the difficulty of achieving sufficient coverage for low-abundance strains (61). Because long-read technologies generally yield lower total sequencing depth than short-read platforms, members of the rare biosphere, which are often the source of ecological recruitment or early evolutionary events, may fail to assemble into contiguous haplotypes. Consequently, this framework is presently most effective for tracking the dominant and moderately abundant lineages within a community. To extend the resolution to rare strains, hybrid approaches could be employed. This involves mapping high-depth longitudinal short reads onto a long-read pangenome graph generated from a deeply sequenced baseline sample (32) or utilizing targeted enrichment strategies prior to long-read sequencing (62, 63).

Third, mapping reads directly onto the pangenome graph can reveal quantitative changes across specific nodes and edges over time. Yet, this presents a computational challenge, particularly in graph regions experiencing overlapping evolutionary events, such as viral integration hotspots. To distinguish multiple structural shifts occurring concurrently, trajectory clustering and long-read phasing could be applied (66). Nodes and edges representing a single evolutionary event, such as a cohesive metabolic cassette sweeping the population, will exhibit correlated coverage trajectories over time. By clustering these co-varying elements, overlapping trajectories can be mathematically resolved into discrete evolutionary events, simultaneously minimizing computational costs. Furthermore, mapping long reads directly to the graph at various time points allows for physical phasing, confirming whether newly acquired nodes reside on the same haplotype background or represent distinct, parallel acquisitions in competing lineages (67). Finally, these structural shifts can be analyzed alongside forward-time simulators (SLiM [64]) to test whether observed node expansions align with neutral dispersal or true positive selection. This approach addresses the limitations of current microdiversity-aware methods; while read-mapping tools like inStrain (32) are excellent for resolving core-genome microdiversity, they remain inherently blind to novel functional insertions if the required reference scaffold lacks those specific accessory nodes.

Furthermore, before inferring selection, defining the standing stock of variation is necessary. Evolutionary inference in open systems ideally requires sequencing of the metacommunity. However, as it is neither practical nor economical to continuously sequence the entire rare biosphere, upstream sources, and sediments, observational time series alone will always contain spatial and temporal gaps. To mitigate this, researchers should employ high-frequency sampling intervals that are shorter than the frequency of the dominant environmental oscillation (e.g., weekly sampling to resolve monthly resource pulses, or capturing specific hydrological events like spring overturn) to prevent the “aliasing” effect. If a lineage (or clone) sweeps locally but is present in the metacommunity, the most parsimonious explanation is ecological lineage turnover driven by dispersal, not evolution. This spatial context must be coupled with temporal realism. By estimating generation times, one can flag “impossible sweeps,” when shifts in allele frequency exceed the maximum theoretical replication rate of the taxon. Rates exceeding this biological limit imply immigration or the reactivation of a dormant, undetectable lineages.

In addition, we rely on forward-time simulation frameworks like Selection on Linked Mutations (SLiM) (64) to serve as a rigorous hypothesis-testing engine against the high-resolution observations (Fig. 2b). As critics rightly note, simulations cannot recover accessory genome information absent from fragmented short-read data. However, when paired with long-read graphs, SLiM allows users to explicitly simulate the unsampleable reservoirs (seed banks and immigrants) that drive ecological turnover. Furthermore, SLiM allows users to program mobile genetic elements with different inheritance rules than the core genome and thus both ecological lineage turnover (e.g., a strain blooming from a simulated seed bank) and true evolutionary adaptation (e.g., a gene sweeping via HGT) can be simulated.

To bridge these complex simulations with empirical data, approximate Bayesian computation (ABC) can be utilized (64, 65). ABC allows the comparison of the simulated scenarios against the actual high-resolution metagenomic time-series (Fig. 2b). This provides a robust statistical framework to quantify whether an observed genomic shift was driven by adaptation or neutral dispersal, estimating evolutionary parameters even when exact mathematical equations are too complex to solve directly.
